# Diseases Admitted under the Care of the Physicians of the Westminster Hospital, from the 23d of July, to the 20th of August, 1800

**Published:** 1800-09

**Authors:** 


					( 201 )
STATE OF DISEASES IN LONDON.
Difeafes admitted under the Care of the Phyficians of the Wejlminfltr
Hofpital, from the 23d (f July, to the 20th of Auguji, 1800.
Continued-Fever - - - 13
Intermictents ----- 1
PJeuri fy ------ 1
Sore Throat - - - - I
Enteritis ------ J
Amenorrhea - ? - 2
Anafarca ----- 3
Afthenia - - - - 9
Catarrh ------ 1
Cholera ------ 4
Cough ------ 4
Diarrhoea - - - - - 4
Eaterodynia ----- 2
Epilepfy ------
Gaftrodynia - - - - -
Hscmorrhois - - -
Hydrops Ovarii - - - -
Hyfteria -------
Jaundice - -
Lepra GrseC - - - - -
Menorrhagia - - - -
Opthalmia - - - - -
Phthifis ------
Rheumatifm - - - -
Vertigo - - - W -
Urticaria - - ? "V ?? -
i
i
2
I
I
I
I
2
2
+
7
i
i

				

